# Role of Submandibular Ultrasound in Airway Management of a Patient With Angioedema

**DOI:** 10.7759/cureus.22823

**Published:** 2022-03-03

**Authors:** Dora H Lin, Brittany Meyers, Saira Nisar, Eric R Heinz

**Affiliations:** 1 Anesthesiology and Critical Care Medicine, George Washington University School of Medicine and Health Sciences, Washington DC, USA

**Keywords:** extubation failure, difficult extubation, difficult airway management, pocus (point of care ultrasound), angioedema

## Abstract

Angioedema is one of several life-threatening clinical scenarios that lacks clarity on when a patient requires intubation. We present a case of angiotensin-converting enzyme-inhibitor-induced angioedema with peri-oral swelling and normal airway measurements on ultrasound, who was intubated with an abundance of caution and extubated successfully. Current tests for intubation and extubation, such as traditional bedside assessments and the cuff leak test, vary in reliability for angioedema and similar urgent situations. Submandibular ultrasound is a quick, low-cost, non-invasive method for determining quantitative criteria for and assessing when intubation and extubation is indicated, which may lead to improved quality of care and patient safety.

## Introduction

Angioedema is a life-threatening condition due to blood vessel dilation and increased vascular permeability that causes swelling of the skin and mucosa [1]. While angioedema can be hereditary or acquired [1], it most commonly leads to emergency department (ED) visits when patients are taking an angiotensin-converting enzyme-inhibitor (ACEi) [2]. Around 0.1%-0.7% of those starting an ACEi develop angioedema [3], which is a substantial number of cases given its widespread use. In 2019, over 25 million patients in the United States filled a prescription for an ACEi [4]. ACEi-induced angioedema can lead to swelling of the face, lips, tongue, larynx, pharynx, and neck, as well as difficulty swallowing, speaking, and handling oral secretions [5,6]. The majority of cases present within the first few weeks after starting use [1].

Currently, endotracheal intubation is often indicated to protect the airway and occurs in 10%-34% of patients presenting to the ED with angioedema [7]. These patients tend to have worsening edema of the oral cavity, pharynx, and larynx and present with drooling, dysphagia, stridor, hoarseness, and dyspnea [8]. However, it may still be unclear at presentation whether a patient requires intubation, and it is often performed out of caution in the event the patient develops respiratory failure. Over the past two decades, there has been a growing interest in the use of point-of-care ultrasound (POCUS) to assess patients perioperatively and in urgent clinical scenarios [9]. Recently, interest has expanded to include using submandibular POCUS to predict difficult airways [10,11]. In this case report, we used submandibular ultrasound to scan the oral cavity prior to intubating a patient with ACEi-induced angioedema.

## Case presentation

**Figure 1:**
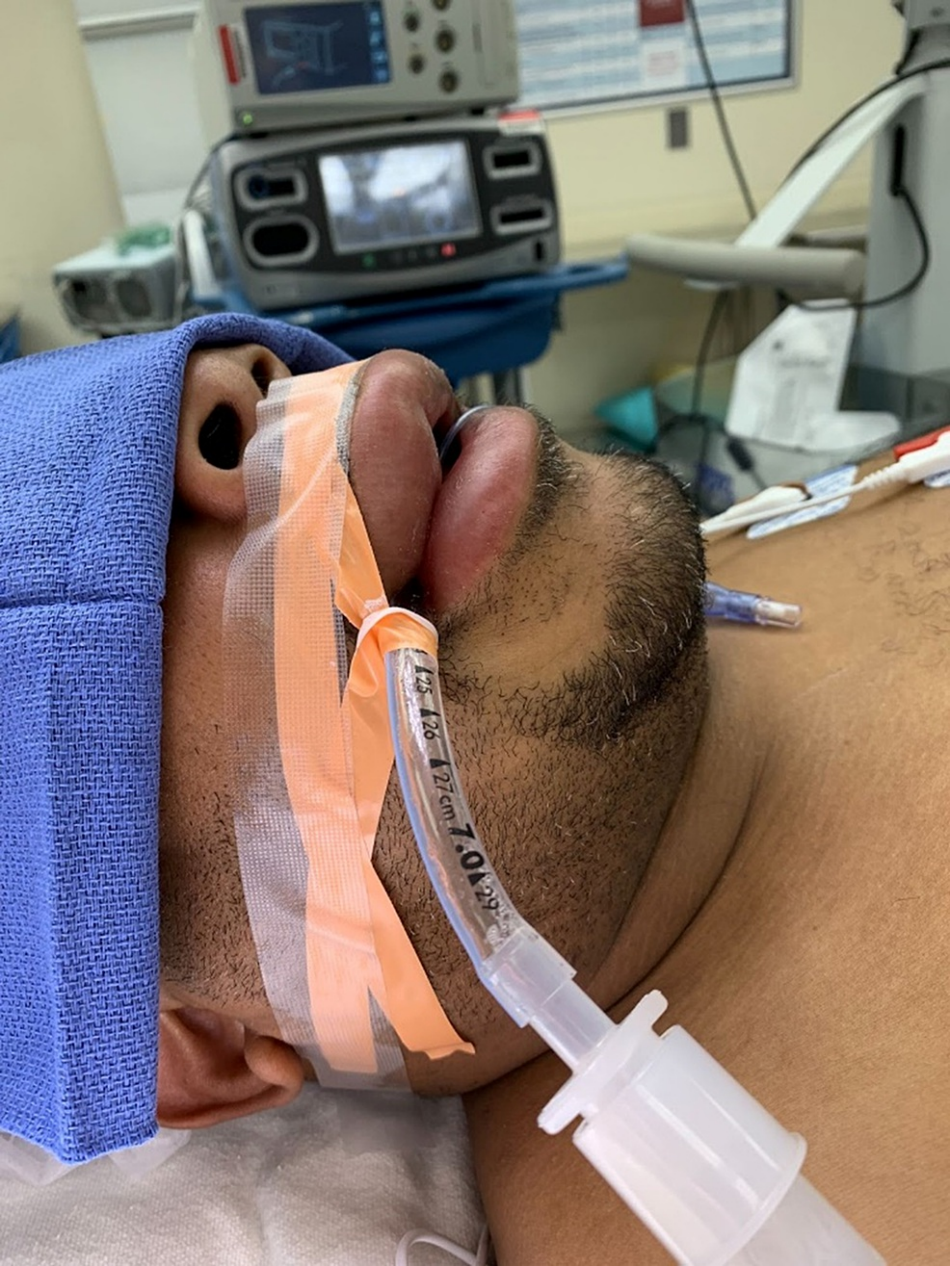
Intubated patient with angioedema of the upper and lower lips.

A 52-year-old African-American obese man presented to the Emergency Department with angioedema (Figure 1). He has a past medical history of hypertension and abdominal aortic aneurysm, with initiation of lisinopril two weeks prior to presentation. He presented with drooling, dysphagia, and sore throat, but denied tongue swelling, cough, or dyspnea.

**Figure 2:**
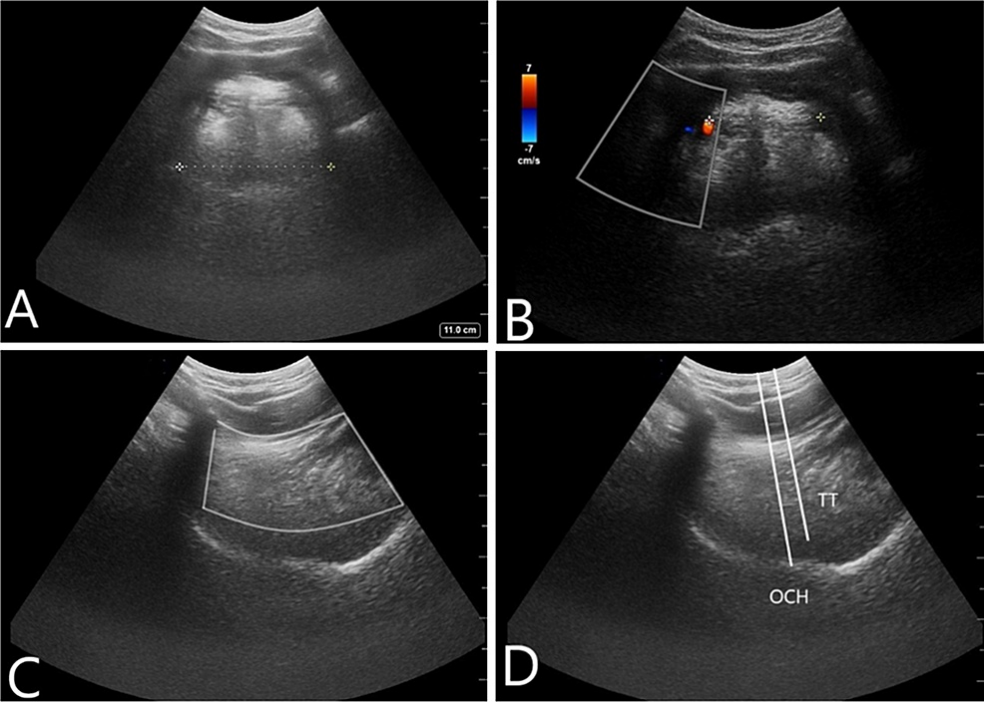
Submandibular ultrasound of the patient’s airway prior to intubation. (A) Tongue width. (B) Distance between lingual arteries. (C) Tongue cross-sectional area. (D) Tongue thickness and oral cavity height. TT: tongue thickness; OCH: oral cavity height.

On physical examination, he was afebrile, normocardic, and hypertensive, with oxygen saturation >95% on room air. He had angioedema of the upper and lower lips, lower face, and eyelid. The angioedema of the lips was primarily right-sided and more upper than the lower lip. Though he had significant angioedema of the lower face, the submental area remained soft. Given that his oral cavity was swollen, traditional bedside airway assessment (including Mallampati score, mouth opening, upper lip bite) was difficult to assess. Therefore, using submandibular ultrasound, we examined the airway and found that the distance between his dorsal lingual arteries was 2.88cm and tongue thickness was 4.76cm (Figure 2A-2D, Table 1).

**Table 1:** Patient’s upper airway measurements using submandibular ultrasound. * Mean and standard deviations previously reported from a few studies, as referenced in this column. Mean (SD) values from Lin et al. (2021) were from those who did not have difficult mask ventilation [11].

	Measurement	Normal Mean (SD)*
Oral cavity height (cm)	5.54	3.65 (0.54) [10]
Distance between lingual arteries (cm)	2.88	2.89 (0.56) [11], 3.04 (0.28) [12]
Tongue thickness (cm)	4.76	3.04 (0.48) [10], 4.43 (0.63) [13], 6.39 (0.51) [11]
Tongue width (cm)	5.21	4.82 (0.50) [12]
Tongue cross-sectional area (cm^2^)	19	
Tongue volume (cm^3^)	98.99	
Tongue thickness to oral cavity height ratio	0.86	0.83 (0.03) [10]

He was given intramuscular epinephrine 0.3mg intravenous (IV), methylprednisolone 125mg IV, and famotidine 20mg IV. Given the patient’s increased drooling, slurred speech, and worsening edema of the upper lip while in the emergency department, consent for intubation was obtained from the patient after explaining the risks and benefits. He was brought to the operating room (OR) for emergent intubation.

Upon arrival in the OR, the patient’s airway was scanned using a SonoSite X-porte Ultrasound system (FujiFilm, Philips Healthcare, Bothell, WA) equipped with a 3 to 8MHz curvilinear transducer. Additionally, he was started on dexmedetomidine infusion for sedation. In preparation for awake fiberoptic intubation, his upper airway was anesthetized with oral 5% topical lidocaine ointment and nebulized 4% topical lidocaine solution. A large base of tongue was visualized without significant compression of the epiglottis. There was no edema of the oropharynx or larynx. The false and true vocal cords were visualized bilaterally with good vocal cord abduction and no sign of subglottic lesions through the vocal cords. Once the fiberoptic bronchoscope was inserted into the trachea and tracheal rings could be identified, a 7.0 cm endotracheal tube was successfully placed without complication. The patient was then transferred to the intensive care unit for further management.

His angioedema was treated with IV methylprednisolone 125mg every 6 hours, oral (PO) famotidine 20mg twice daily, and IV diphenhydramine 25mg every 6 hours. He was sedated on IV propofol (70mcg/kg/min) and IV dexmedetomidine (0.4mcg/kg/hr). Angioedema of the lips and lower face improved by the morning of hospital day 3. The patient passed the cuff leak test and respiratory rate-tidal volume ratio test and was subsequently successfully extubated to nasal cannula on hospital day 3. The patient gave written and verbal consent to publish this de-identified case report.

## Discussion

We present the case of a patient with ACEi-induced angioedema who was emergently intubated in the OR. The decision to intubate was made due to his clinical symptoms which include drooling, worsening oral cavity swelling, and dysphagia - all of which have been found in patients with angioedema to be associated with the need to intubate [8].

Generally, it is difficult to assess the necessity to intubate patients with angioedema, given that not all patients with angioedema have airway compromise and require intubation. A few different approaches for deciding whether to intubate include identifying locations of angioedema and presenting symptoms strongly associated with resulting intubation, as well as determining staging criteria [6,8,13]. However, the proposed staging criteria for angioedema have been based on visual assessment of the upper airway [13]. According to one staging criteria, our patient would be categorized as stage 2 angioedema due to edema of the soft palate, a stage where none of the patients were intubated in this study [12]. Even for more severe stages, less than a quarter of stage 3 and less than half of stage 4 patients required an airway intervention [13]. As such, it is possible that quantitative measurements, like those offered by submandibular ultrasound [10-12], could help elucidate clearer thresholds for intubation.

In our patient, we found that swelling of the oral cavity precluded proper airway assessment using traditional bedside means such as Mallampati score, mouth opening, and upper lip bite. These bedside clinical tests have poor sensitivities and fair specificities, which have been shown to improve with the addition of ultrasound imaging [14]. Thus, we used submandibular ultrasound to further assess his airway. To our knowledge, POCUS in the ED has been reported only once before as having been used for a patient with angioedema [15]. In this previous case, the authors used anterior neck ultrasound to measure laryngeal edema, while we used submandibular ultrasound to measure tongue and oral cavity swelling [15].

On ultrasound, we found the distance between the patient’s lingual arteries (DLA) to be 2.88cm and tongue thickness to be 4.76cm, which are within normal [11,12]. Increasing DLA has previously been reported to be strongly correlated with increasing severity of obstructive sleep apnea, a known cause of difficult intubation [12]. Interestingly, one study of 41 patients reported that tongue base thickness, rather than DLA, is correlated with difficult mask ventilation [11]. These findings suggest that multiple measurements via submandibular ultrasound could be used to determine if intubation is needed and should be further explored. However, we note that the mean tongue thickness was different across previous studies using submandibular ultrasound (Table 1), which may be due to interoperator variability and different methodology used to take measurements [10-12]. Refining the method of submandibular ultrasound measurements can help decrease such variability.

Similarly, it can be difficult to determine when to extubate a patient with angioedema - namely when there is no obstruction of the upper airway [16]. A handful of clinical criteria are currently used to predict the safety of extubation, including the cuff leak test, respiratory rate-tidal volume ratio, and bedside direct laryngoscopy. We decided to extubate our patient after 36 hours, which is close to the average intubation duration in angioedema [16]. He had a negative cuff leak test and sufficiently low respiratory rate-tidal volume ratio (RVR). Moreover, we treated our patient with famotidine and patients with angioedema treated with H1-blockers have been found to be extubated earlier [6]. Our patient was successfully extubated.

However, while a positive cuff leak test may be helpful for identifying airway obstruction, a wide variety of criteria have been used for what is considered positive [17]. Furthermore, findings on whether a negative cuff leak test is reliable for extubation have varied greatly [18,19]. With regards to the RVR, it is a reliable test to reflect adequate respiratory muscle strength and endurance to wean from mechanical ventilation [20]. Nonetheless, intubation for ACEi-induced angioedema is related to upper airway edema and obstruction, instead of respiratory spontaneity or muscle strength. Direct laryngoscopy is used for directly visualizing the airway yet is invasive and very noxious to the patient.

We posit that submandibular ultrasound can be a highly beneficial, non-invasive method for deciding not only when to intubate, but also when to extubate the patient with angioedema. As ultrasound is becoming more available and utilized in perioperative care, this modality is limited by provider training, experience and comfort level. In retrospect, we could use ultrasound prior to extubating the patient to visually and quantitatively examine airway edema. Given the current uncertainty around airway management in upper airway obstruction, submandibular ultrasound should be further investigated for urgent clinical settings, such as angioedema.

## Conclusions

Submandibular ultrasound is a quick, low-cost, non-invasive imaging modality that may improve quality of care by determining whether a patient needs an airway intervention in unclear clinical presentations, such as angioedema. Our case is an example of a situation that could benefit from the quantitative measurements that submandibular ultrasound can offer, in combination with developing clearer thresholds for intubation and extubation. Future studies should explore the inclusion of submandibular ultrasound measurements as part of the clinical indications that guide urgent clinical scenarios with upper airway obstruction.
